# Negative Pressure Wound Therapy Assisted Closure: An Effective Mode of Management for Infected and Contaminated Wound With Non-Union Fracture Femur

**DOI:** 10.7759/cureus.9037

**Published:** 2020-07-07

**Authors:** Bishnu P Patro, Susanta Khuntia, Nabin K Sahu, Gurudip Das, Saroj K Patra

**Affiliations:** 1 Orthopaedics, All India Institute of Medical Sciences, Bhubaneswar, IND; 2 Trauma & Orthopaedics, All India Institute of Medical Sciences, Bhubaneswar, IND

**Keywords:** wound closure, infection, non-union, femur and fracture, negative pressure

## Abstract

High-energy open fractures are often associated with significant soft tissue damage and can have contamination. Infection of a fracture can be the most detrimental factor for fracture union. Control of infection and soft tissue coverage over exposed bone plays a vital role in its overall outcome. Negative pressure wound therapy (NPWT) assisted closure has depicted encouraging results for helping control of infection and wound closure. NPWT assisted closure promotes reduction of bacterial load in the wound, facilitates removal of secretion from the wound, promotes the formation of granulation tissue, and decreases wound size. We present a case of open fracture femur with severe infection and exposed bone. Along with infection and comminution of fracture, there was collection of necrotic tissue at the fracture site. Infection settled with debridement of wound and application of NPWT. With the application of NPWT, there was formation of granulation tissue and a decrease in wound size. The wound healed completely following application of secondary sutures. Any form of plastic procedures, such as muscle pedicle graft and split-thickness skin grafting, was not required for wound closure. NPWT-assisted closure is a promising mode of wound management in grossly infected wounds and obviates the need for further plastic procedures. The effect can be extrapolated to all open wounds with infection but must follow a thorough debridement and lavage.

## Introduction

A comminuted fracture of long bones is associated with significant soft tissue damage. The fracture can be simple or open in nature, depending on the mode of injury. Younger males are mostly affected by motor vehicle accidents [1]. In developed countries, the annual incidence of midshaft femur fracture is about 10 per 100,000 population [2]. In developing countries, the incidence is far more and is about 15.7-45.5 per 100,000 population per year [3]. Open fractures depending upon the degree of infection can be managed either by internal fixation or a staged procedure after application of external fixation. In many places, internal fixation is considered if the surgery is performed within 24 hours of injury or when there is less contamination. Infection following intramedullary nailing is an extremely morbid complication that is challenging to manage [4]. The most common organism isolated in this infection is Staphylococcus aureus [5]. With infection, patients usually present with fever, pain, and other local signs of inflammation. Occasionally, they present with abscess or a draining sinus. Clinical suspicion of infection is the first diagnosis in such a scenario. There may be raised erythrocyte sedimentation rate (ESR), C-reactive protein (CRP), and total leucocyte count (TLC). The organism can be isolated by the culture of infected tissue or discharge from the wound [6]. Finally, the patient ends up with two problems, i.e., infection and non-union of the fracture. Infected non-union with bone defect following intramedullary nailing is well managed with bone transport methods [7]. Condition of the patient is complicated by the infected wound and exposed bone ends, which will need multiple plastic procedures to cover the bone. Negative-pressure wound therapy (NPWT) is a novel method to tackle complicated wounds with infection and non-union [8,9]. It helps in increasing blood flow, angiogenesis, decreasing wound size, and inducing cell proliferation; as a result, it helps in early wound closure [10]. Besides, NPWT promotes the growth of granulation tissue and union at the docking site following bone transport in infected non-union [11]. NPWT is useful for patients having infection and discharge from the fracture site. We present a case of open femur fracture with severe infection and exposed bone following fixation with an intramedullary nail and managed with NPWT and bone transport.

## Case presentation

**Figure 1:**
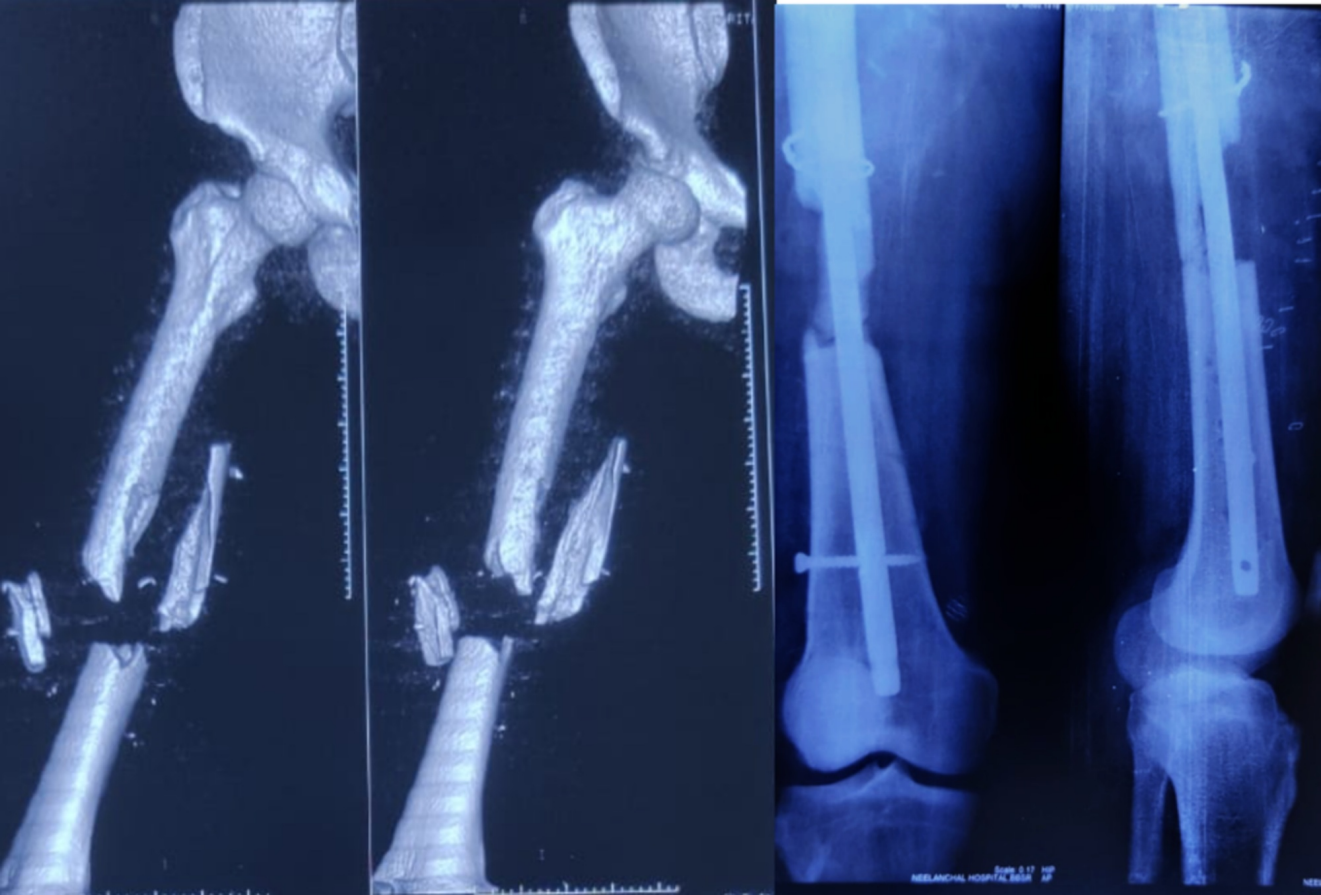
Comminuted femur fracture, loss of bony fragment, and Interlocking nail in situ.

**Figure 2:**
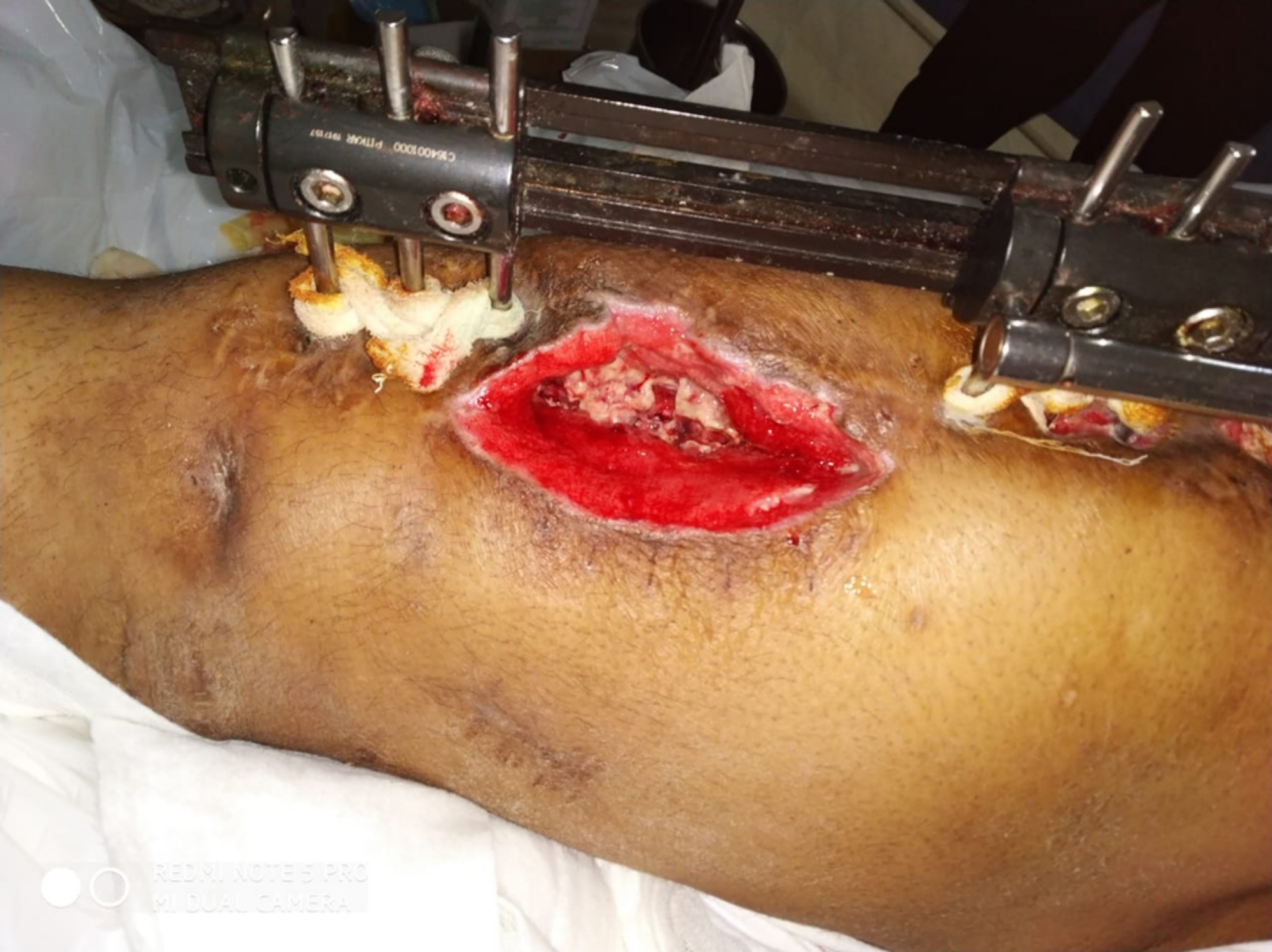
Wound with necrotic tissue (white tissue).

**Figure 3:**
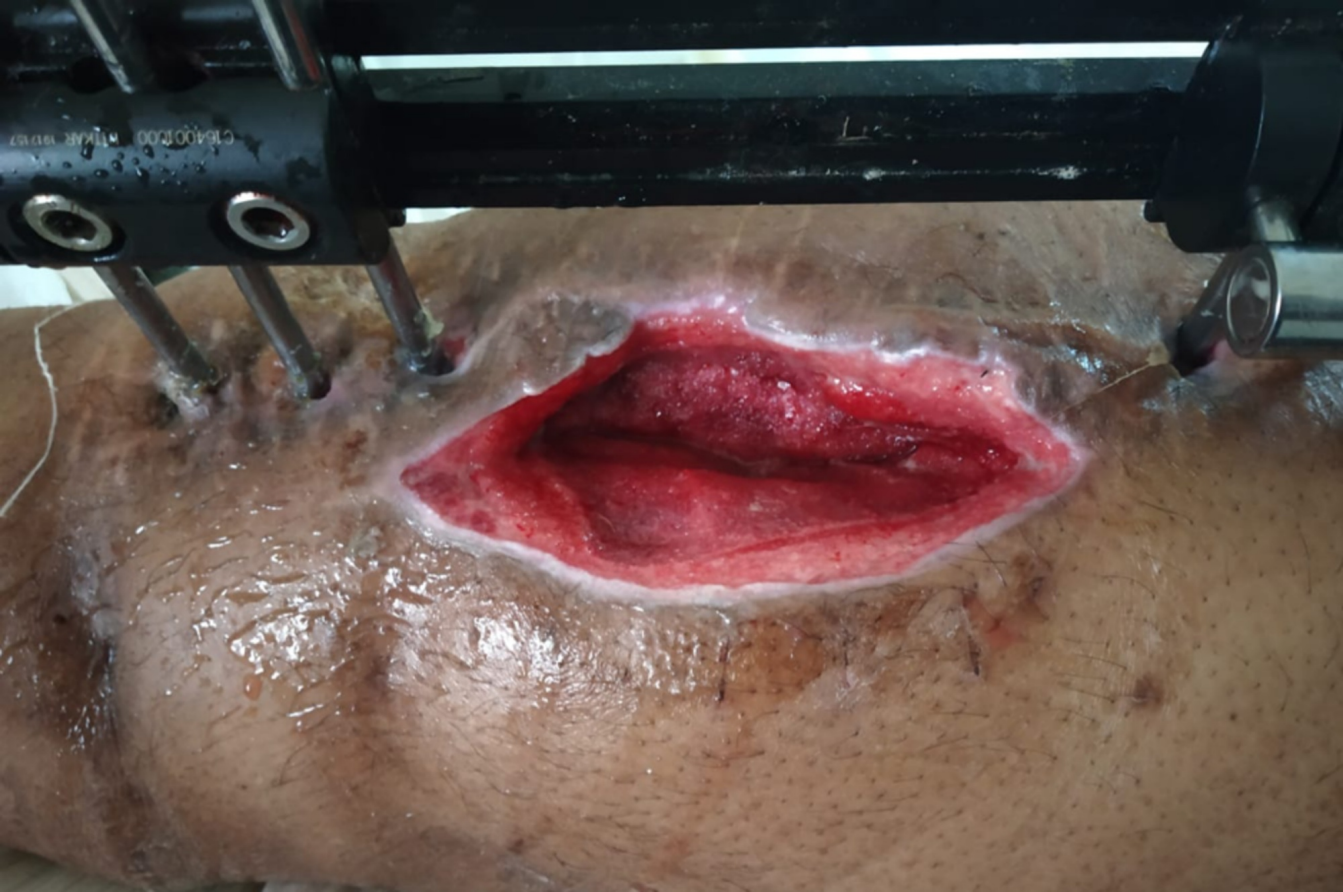
Clean wound after the use of negative-pressure wound therapy.

**Figure 4:**
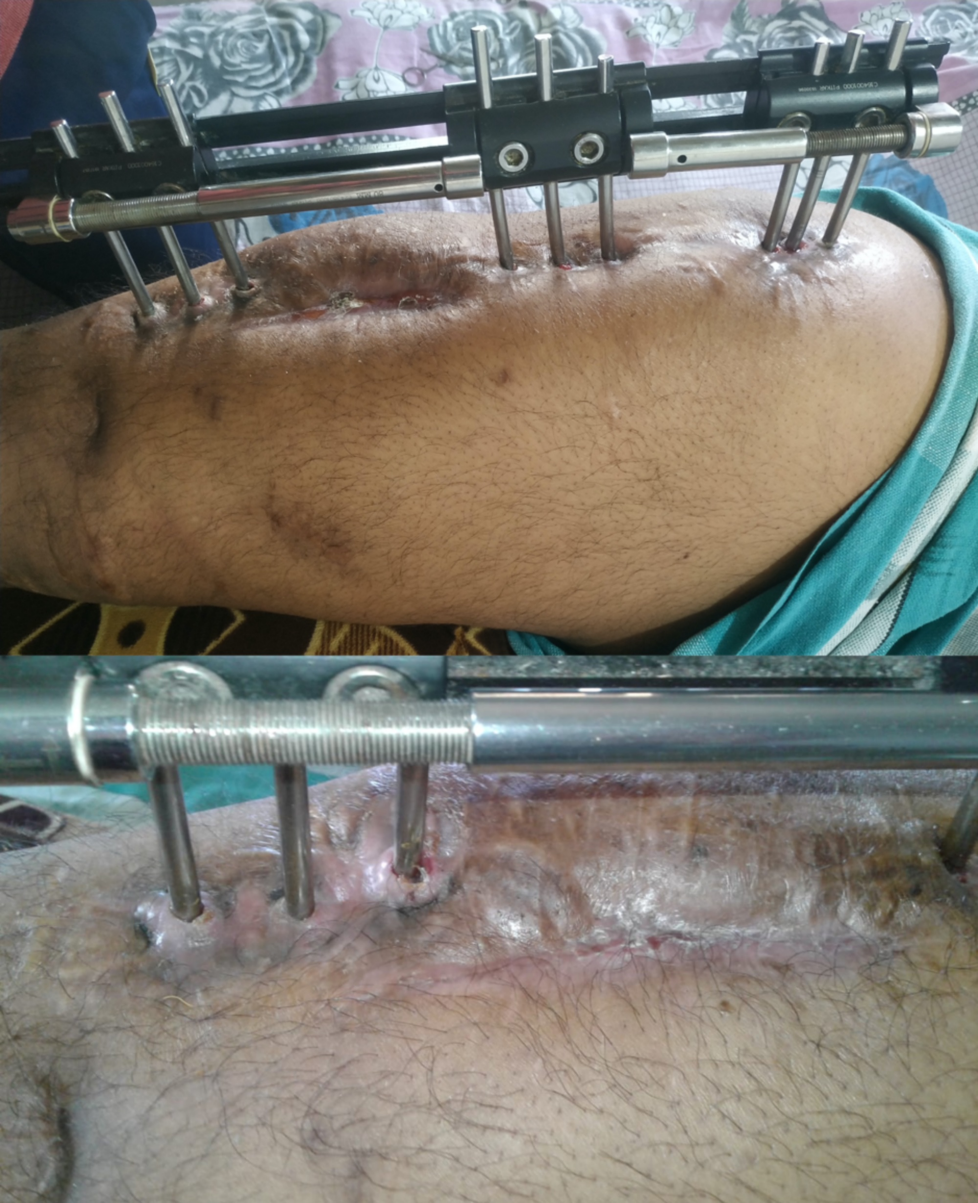
Healed wound.

**Figure 5:**
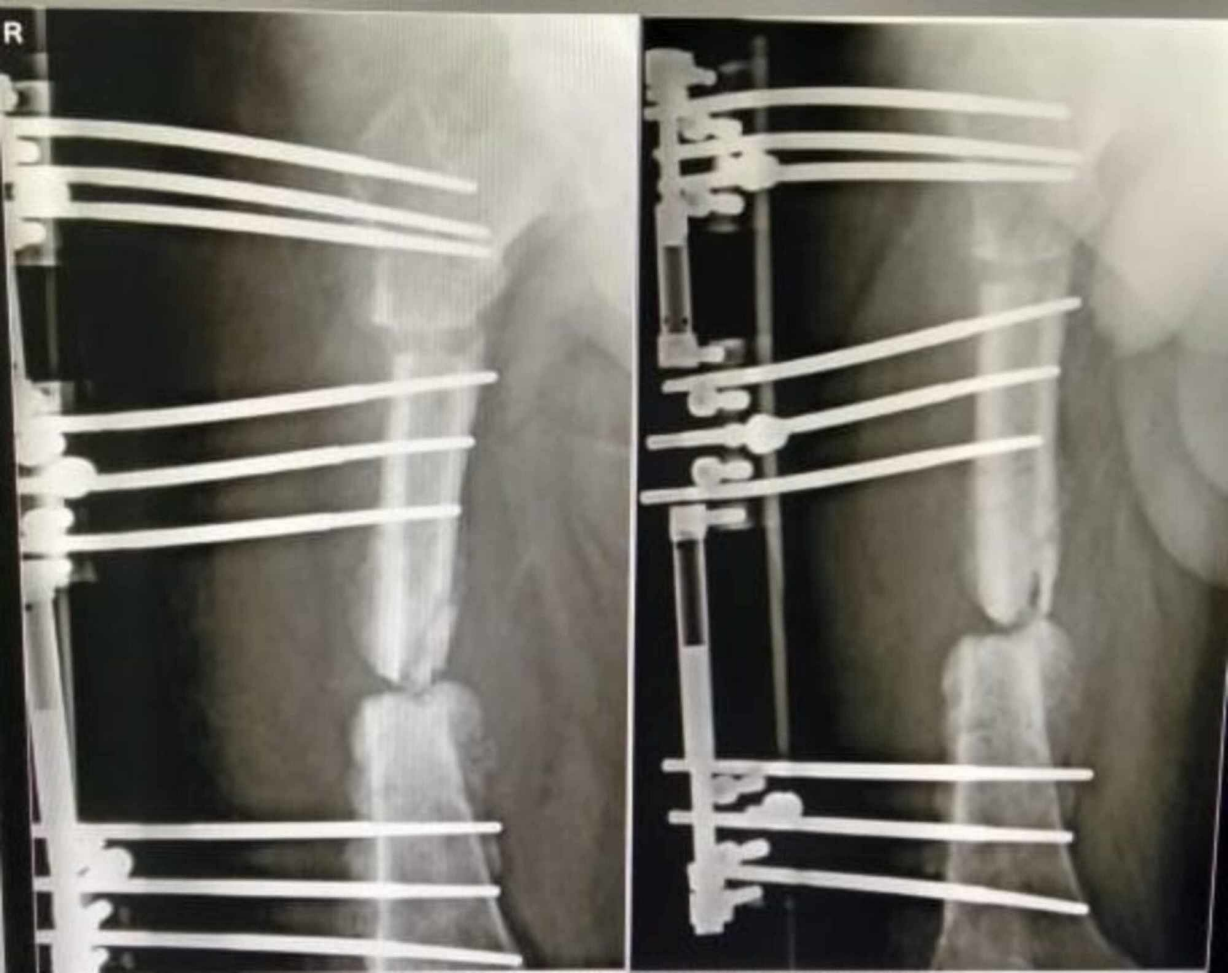
Union in progress.

A 32-year-old male sustained a grade 3B (Gustilo and Anderson) open fracture of the right femur following a high-speed motor vehicle accident. A piece of bone from the femur was lost at the site of injury. He did not have any other injury except fracture of the femur. At the primary center, he received wound lavage and splinting of the limb along with systemic antibiotics. Due to lack of facility at the primary center, the patient was referred to the secondary center. After five days of index injury, the surgeon in the secondary hospital performed surgical debridement of the wound and applied external fixator. Culture reports and antibiotic profile of the index surgery are not available due to lack of documentation. The wound healed satisfactorily, and stitches were removed in two weeks. After 15 days of the first surgery (20 days from injury), the operating surgeon removed the external fixator and did intramedullary nailing of the femur without bone grafting (Figure 1). The incision wound healed with routine removal of skin stitches. After three months of intramedullary nailing of the femur (nearly four from injury), a blister developed in the lower thigh, which later burst with purulent discharge. The same surgeon after culture and sensitivity of pus administered systemic antibiotics and the discharged decreased after 20 days of antibiotics. After 15 days (five months from injury), the patient presented to another surgeon with similar discharge and was administered empirical systemic antibiotics for a period of 30 days. The patient had marginal improvement with occasional fever and knee pain. Following intramedullary nailing, no further wound washout or debridement was performed by earlier surgeons. At this point (nine months after injury), the patient presented to me with complaint of pain knee, unable to walk, and discharge from the distal thigh. The patient was more than 100 kg with a BMI of 32 kg/m2 and had treatment of more than nine months with a significant psychological aspect. At this point in time, his blood parameters were within normal limits ( TLC was 12.29 x 103/cumm, neutrophil count was 78%, ESR was 6 mm per one hour, CRP was negative, and pus culture revealed no growth). The infection was not revealed due to long-term administration of antibiotics. In our first stage (nine months and 15 days after injury), we removed the intramedullary nail and put a limb reconstruction system (LRS) after thorough debridement. We removed the dead part of the bone nearly 40 mm and put vancomycin cement bead chain. Pus collected from the deeper part of the wound collected during debridement revealed Escherichia Coli and was only sensitive to colistin. The patient was treated with intravenous colistin for two weeks with regular dressing of the wound. There was no improvement in wound even after two more debridements. We had to remove the antibiotic cement bead (10 months and 10 days after injury) and found a lot of necrotic tissue at the fracture site (Figure 2). After another debridement (10 months and 15 days after injury), we put an NWPT dressing at an intermittent pressure of -120 mm Hg. Surprisingly, within 72 hours, there was a significant improvement in the wound in terms of collection of debrima, wound margins, and so on. The dressing was changed every 72 hours, and after three dressings we found a healthy wound with clean wound margin and formation of granulation tissue (Figure 3). We were able to close the wound with secondary suture (11 months and five days after injury) as the wound gap decreased with NPWT. The wound healed completely within three weeks (12 months after injury) without any further discharge (Figure 4). The patient was put on suppressive antibiotic therapy, and the bone defect managed with corticotomy and bone transport on an LRS (Figure 5) (union in progress).

## Discussion

The patient in the discussion had a significant infection following intramedullary nailing of the femur with collection of profuse necrotic materials. The surrounding muscle tissue was not healthy looking. There was no remarkable improvement with routine dressing even after repeated debridement. With the use of NPWT, the necrotic tissue load decreased significantly, and there was formation of granulation tissue and decrease in the size of the wound. Muscles turned red within five days of NWPT. The wound healed nicely with secondary sutures and without any further need of muscle or skin flap. It can be presumed that regular dressing might not have reduced the wound size and that would have warranted need of flap cover. NPWT has delivered appreciable results in terms of wound healing in this grossly contaminated and infected wound.

Yadav et al. have found better results with NPWT in managing chronically infected wounds compared with conventional technique [12]. Cozza et al. conducted a prospective experimental study including more than 10 patients and found that NPWT is not a risk factor for health care personnel associated with the management of infections [13]. Arundel et al. in their randomized control study comparing NPWT with routine wound management found that healing of surgical wounds by secondary intention is very difficult. There is little evidence of one method being better over others in the treatment of these complex wounds. But NPWT may be a cost-effective method of wound management [14]. Huang et al. suggested that a proper understanding of mechanobiology, biofilms, and cell therapy is essential in the better management of complicated wounds [15]. Itani found NPWT an easy solution for closed orthopedic incisional wound complications such as hematoma, seroma, infections, and wound dehiscence [16]. Zhou et al. showed that NPWT with -75 mm Hg pressure has a similar effect on bacterial load reduction and wound healing as that of NPWT with higher negative pressure. Moreover, comparing with high negative pressure, relatively moderate negative pressures contribute to better wound healing through accelerated granulation tissue formation, increased angiogenic factor production, and improved collagen fiber deposition. Increased microvessels were generated in wounds treated by NPWT using -75 and -150 mm Hg pressure compared with that using -225 and -300 mm Hg on days 3 and 5, respectively [17]. In our study, we adopted a pressure of -120 mm Hg and found appreciable results in terms of the formation of granulation tissue and wound healing. Janssen et al. in their systematic review observed decreased quality of life and increased anxiety of patients during NPWT for the management of infected wounds [18]. As patients have to undergo multiple surgeries and remain bed-bound for a longer period, they develop depression besides anxiety. Li et al. found that if the NPWT is applied early in an infected wound, it can prevent the formation of Staphylococcus aureus biofilm [19]. Hou et al. assessed 32 Gustilo type IIIB open tibia fractures and found that NPWT reduces the flap size required to cover the wound. They also found that there is an increase in infection if the NPWT is used for more than seven days [20]. In our study, wound healed with secondary suture, and the need for flap cover was avoided with the use of NPWT.

In our patient, we had encouraging results of NPWT use as that of studies by other scholars.

## Conclusions

NPWT-assisted wound closure is a magnificent tool for wound management. We had encouraging results with the use of NPWT in wound management. It is essential to have a thorough debridement of the wound before the application of NPWT and regular change of dressing every 48 hours to 72 hours as per the volume of secretion to prevent secondary infection and have a better outcome. In addition to NPWT, we would suggest having sequential concentric compression of the limb distal to proximal to encourage venous return as most of these patients have poor muscle pump. The effect can be extrapolated to all wounds with infection and discharge but must follow a thorough debridement and lavage.
